# Inflammation in acute coronary syndrome: Expression of TLR2 mRNA is increased in platelets of patients with ACS

**DOI:** 10.1371/journal.pone.0224181

**Published:** 2019-10-23

**Authors:** Lukas Andreas Heger, Marcus Hortmann, Madlin Albrecht, Christian Colberg, Karlheinz Peter, Thilo Witsch, Daniela Stallmann, Andreas Zirlik, Christoph Bode, Daniel Duerschmied, Ingo Ahrens

**Affiliations:** 1 Department of Cardiology and Angiology I, Heart Center Freiburg University, Faculty of Medicine, University of Freiburg, Freiburg, Germany; 2 Baker IDI Heart and Diabetes Institute, Melbourne, Australia; 3 Department of Cardiology, Medical University of Graz, Graz, Austria; 4 Department of Cardiology and Medical Intensive Care, Augustinerinnen Hospital, Academic Teaching Hospital University of Cologne, Cologne, Germany; Medizinische Hochschule Hannover, GERMANY

## Abstract

**Background:**

Platelets are key components in atherogenesis and determine the course of its clinical sequelae acute coronary syndrome (ACS). Components of the innate immune system—the superfamily of TLR receptors–are present in platelets and represent a link between atherothrombosis and inflammation. We hypothesize that alteration in platelet TLR mRNA expression is a result of inflammation driving coronary atherosclerosis and may represent an alternative platelet activation pathway in ACS.

TLR2-, TLR4- and TLR9- mRNA-expression was determined in ACS patients and compared to patients with invasive exclusion of atherosclerotic lesions of coronary arteries.

**Methods:**

A total of fifty-four patients were enrolled in this clinical retrospective cohort single centre study. Total RNA from sepharose-filtered highly purified platelets was isolated using acid guanidinium thiocyanate-phenol-chloroform extraction and transcribed to cDNA using a first strand cDNA synthesis kit. To determine absolute copy numbers of TLR2, TLR4 and TLR9 we used plasmid based quantitative PCR with normalisation to an internal control.

**Results:**

We found that mRNA expression levels of TLR2 but not TLR 4 and 9 are up-regulated in platelets of patients with ACS when compared to patients without coronary atherosclerosis.

**Conclusion:**

Our results suggest elevated TLR2 mRNA expression in platelets as a biomarker reflecting the underlying inflammation in ACS and possibly severity of coronary atherosclerosis. Platelet TLR2 may represent a link between inflammation and atherothrombosis in ACS.

## Introduction

Platelets play a crucial part in genesis and course of acute myocardial infarction (AMI). [1, 2] As part of the innate immune system, they facilitate the recruitment of inflammatory cells to lesion sites in vessels, promoting endothelial dysfunction and initiating atherosclerosis-formation. [3, 4] Following atherosclerotic-plaque rupture or fissure, platelets aggregate and conduce to the formation of an unstable thrombus potentially leading to reduced coronary flow or distal embolization. [5]

Some of the platelets functions are exerted via the secretion of cytokines [6, 7] while others are transferred through expression of surface proteins / receptors. [8, 9]

Although platelets belong to the anucleate cells, they have the potential to synthesize proteins to exercise their effect in response to physiologic stimuli. [10] They are equipped with all organelles necessary for protein synthesis on basis of translation from megakaryocyte derived mRNA named, the platelet transcriptome. [11],[12, 13],[14] This strictly post-transcriptional control enables platelets to act rapidly upon activation. [6]

Apart from a select group of mRNAs, which are translated into proteins on a regular basis, platelets also contain unprocessed pre mRNA transcripts and are equipped with a functional, activation-dependent spliceosome that converts these transcripts to translatable mRNAs. [15, 16] This platelet pre mRNA enables platelets to enlarge their amount of secretable proteins upon corresponding stimulation. [17, 18]

Recent exploratory analysis of the platelet transcriptom confirmed ACS-specific gene expression patterns suggesting platelet mRNA expression as a possible marker in ACS. [19] The study at hand hypothesizes that alteration in platelet mRNA expression could be of use in ACS diagnostic.

Subject of our mRNA analyses were expression levels of members of the superfamily of Toll-like-receptors (TLR) in platelets. These membrane-spanning proteins orchestrate platelet function upon activation, partly through increased splicing and translation to protein of platelet pre mRNA. [20–23] Due to their role as mediators of a thromboinflammatory response and in being a possible link between both platelet-function and the innate immune system, TLRs are vividly focused on by research regarding their role in ACS. (24-[24–26]

TLRs, as part of the innate immune system, detect molecular patterns associated with a variety of exogenous pathogens (PAMPs) and markers of endogenous damage (DAMPs) mediating platelet response accordingly [27, 28]. To date 7 of the reportedly 11 members of the TLR-family expressed in humans are found to be expressed on platelets (TLR1, TLR2, TLR4, TLR6, TLR7, TLR8, and TLR9) [29] with each one recognising different DAMPs and PAMPs and consecutively triggering its own distinctive biological response. [30, 31]

Up to date, TLR expression is believed to be a remnant from the megakaryocyte and hence product of platelet ontogeny. [32, 33]

Nevertheless, recent studies provide evidence that by presenting processed pathogens via TLR receptors to members of the adaptive immune system platelets seem to link adaptive and innate immune response [34].

However the exact intracellular signal pathways of the TLRs are yet to be defined. Up to date TLRs are thought to form homodimers -or in case of TLR-2 heterodimers- with TLR-1 and TLR-6 [35] to assembly, through a complex cascade, a multiprotein signalosom leading to the activation of transcription factors advancing the expression of an array of inflammatory genes. [23, 36]

Elevated expression levels of TLRs are reported in atherosclerotic lesions, macrophage-infiltrated coronary artery plaques obtained at autopsy and in circulating monocyte cells of patients with ACS. [36, 37]

Also recent epidemiological studies associate TLR-expression patterns with distinct cardiovascular risk factors such as diabetes and high blood pressure and suspected them to account for screening relevant sex differences. [30]

Furthermore, TLR activation through local release of DAMPS during ACS is reported to aggravate myocardial ischemia/reperfusion (I/R) injury. [38, 39]

When activated, platelets boast with increased intracellular expression of TLR 2, TLR 4 and TLR 9, and expression rates reportedly correlate with platelet activation.[8, 40]

Amongst the different TLRs, Toll-Like receptor 4 is the most prevalent expressed TLR on platelets. [41] Both TLR 2 and TLR 4, upon activation, advance the immune response and exercises pro-thrombotic functions via increased platelet thrombin generation. [42] [22, 43, 44] Also recent studies single out TLR-4 to be an important player in the initiation and progression of atherosclerotic disease as in a mouse model the lack of TLR 4 reduced atherosclerosis lesion sites. [45–47]

Despite a high sequence homology and supposedly similar cytoplasmic signalling programs of TLR2 and TLR4, several studies reveal differences in their final effect. [48, 49]

Their exact role in ACS is yet to be established.

In the case of TLR2, recent data additionally suggests a further form of function in, so called, soluble TLR2 receptors. [50] The Soluble TLR2 receptor is reportedly generated from a post-translational modification in the TLR2 protein and retained and distributed by macrophages. [50] Following plasma bacterial lipopeptide exposure, sTLR2 is believed to encapsulate bacterial lipoproteins acting like a decoy receptor. Hence sTLR2 slows down pro-inflammatory cytokine production. [51] There is evidence of alteration in sTLR2 levels in pathogenesis in post Myocardial infarction heart-failure. Whether there is an active role in ACS of sTLR2 is yet to be established. [52]

TLR9 is located intracellular in the endosome compartments. [31] Preclinical studies report that Toll like receptor 9 activation can also be pro-thrombotic. [53, 54] However TLR 9 function is yet controversial with evidence of TLR 9-mediated aggravation of I/R injury and ameliorating cardiac rupture after ACS. [55, 56]

In the study at hand we hypothesize that platelet TLR mRNA expression may represent an alternative platelet activation pathway in ACS. In a subset of patients with myocardial infarction we assessed -using absolute plasmid based quantitative Real Time PCR—the mRNA levels encoding for TLR receptors 2,4 and 9 in highly purified platelets from patients with ACS and compared them to TLR mRNA expression levels of patients without coronary artery disease. In a subset analyses we differentiate between myocardial infarction with ST-Segment-Elevation (STEMI) and without (NSTEMI). This aims to further understand TLR-receptor regulation in platelets and to elucidate the importance of TLR-receptor in the pathogenesis of myocardial infarction.

Since soluble TLR2 (sTLR2) reportedly interacts with TLR 2 function, in the sense of being a negative regulator of TLR-signalling, this study also investigated–using an Enzyme-linked immunosorbent assay (ELISA)- plasma levels of sTLR2 in patients with NSTEMI, STEMI and Non-CAD. [51] Studies describe an sTLR-mediated immune response especially in bacterial and viral infections. [57] We argue that similar patterns could be the case in ACS.

## Material and methods

### Subjects

Fifty-Four subsequent patients admitted to the Department of Cardiology and Angiology I of the University Heart Center in Freiburg for acute or elective coronary angiography were included in this study: 26 patients with ST-segment elevation myocardial infarction (STEMI), 14 patients with non-ST-segment elevation myocardial infarction (NSTEMI) and 14 with normal epicardial coronary arteries in the coronary angiography defined as the non-coronary-artery-disease group (non-CAD).

The protocol of this study conforms to the ethical guidelines of the 1975 Declaration of Helsinki and was henceforth approved by the institutional ethical committee of university of Freiburg (permit numbers EK57/06 and EK 379/09). Written informed consent was obtained from all patients.

STEMI and NSTEMI were defined according to the recent guidelines published by the European society of cardiology.

Patients were recruited from 2010–2013.

Peripheral blood vessels samples of STEMI and NSTEMI patients were collected within 24 hours of confirmed diagnosis. Exclusion criteria were inflammatory diseases such as rheumatoid arthritis or sepsis according to the guidelines [58, 59]. Also excluded were patients suffering from tumour disease or showed clinical signs of infection (fever) and/or did need antibiotic treatment at the time of sample collection.

### Patient characteristics

All patient clinical characteristics were obtained retrospectively from the hospital's electronic database. Pharmacologic treatment before and after PCI was conducted according to the European Society of Cardiology (ESC) Guidelines. Maximum levels of all laboratory values, including levels of Thrombocytes, Leukocytes, Haemoglobin, Creatinkinase, Troponin T and CRP within 24 h of the ischemic event were recorded and used for statistical analysis.

### Primary percutaneous coronary intervention (PCI)

PCI was performed with standard catheters. All STEMI and NSTEMI patients received aspirin (minimum of 250 mg) and an ADP receptor blocker (prasugrel 60 mg, ticagrelor 180 mg or clopidogrel 600 mg). Unfractionated heparin (5000 U) was administered prior to angiography. None of the patients included in the study received bail-out therapy with GPIIb/IIIa inhibitors.

### Purification of platelets

Upon inclusion of study participants, 40 ml blood was drawn from a peripheral vein into polypropylene sodium-citrate-coated tubes *(Thermo Fisher Scientific Inc*., *Waltham*, *Massachusetts*, *USA)* and processed immediately. Whole blood was centrifuged at 150G using Multifuge 3S *(Heraeus*, *63450 Hanau*, *Germany)* for 15 minutes to obtain Platelet-rich plasma (PRP) via density gradient variations of blood ingredients. Consequently the whole blood separates into three layers: an upper layer containing mostly platelets and white blood cells, an intermediate thin layer known as the buffy coat rich in white blood cells, and finally a bottom layer that consists mostly of red blood cells. The upper layer was then carefully aspirated and afterwards put to further use. In a second purification process size exclusion chromatography was used to elute contamination from PRP. PRP was pipetted correspondingly on 4ml sepharose *(CL2B300; Sigma-Aldrich*, *St*. *Louis*, *Missouri United States)* each filled into 6 disposable filter columns *(Thermo Fisher Scientific Inc*., *Waltham*, *Massachusetts*, *USA)*. Filter columns were washed with JNL buffer twice before use to eliminate ethanol. JNL Buffer was prepared freshly from stock solution for all experiments according to the following protocol:

JNL A: 60mM Dextrose, sterile-filtered
JNL B: 1.3M NaCl, 90 mM NaHco_3_, 1M Na_3_C_6_H_5_O_7_, 100mM Tris base, 30mM KCl
JNL C: 20mM Hepes
JNL D: 8.1mM KH_2_PO_4_
JNL E: 90mM MgCl_2_6H_2_O

10 ml JNA A, JNL B, JNL C, JNL D and 1ml of JNL E were mixed and adjusted to pH 6,5 using Acid Citrat Dextrose (ACD: 38mM C_6_H_8_O_7_, 75 mM Na_3_C_6_H_5_O_7_, 136mM C_6_H_12_O_6_, sterile filtered). Finally 10ml of 100mM Theophyllin and Millipore filtered water was added to the solution up to match 100ml.

PRP was allowed to sink through the sepharose-filled columns with 1ml of JNL Buffer. According to molecule size limiting migration speed, the first drops eluting from the tip of the filter columns clear, consisting mainly of proteins.

Drops containing platelets however, were cloudy and collected in a 15ml falcon. Purified platelets were hence again washed with 10ml JNL buffer and pelleted by centrifugation at 500G. After discard of the supernatant, the pellet was thoroughly re-suspended and again washed twice by re-suspension in 1ml JNL buffer and corresponding centrifugation at 2000G.

Finally the pellet was dissolved in 6ml of JNL buffer and carefully layered on a discontinuous Density gradient medium (OptiPrep; STEMCELL Technologies Inc., Vancouver, British Columbia, Canada) according to the manufacturers guidelines followed by density gradient centrifugation at 300G for 20 minutes. After centrifugation, platelets are represented in the middle phase cell rich halo and carefully aspirated and diluted in 15ml JNL buffer with 1mM Theophyllin. The suspension was then centrifuged at 1000G for 10 minutes. The emerging platelet pellet was dissolved in 1ml TRIzol *(Thermo Fisher Scientific Inc*., *Waltham*, *Massachusetts*, *USA)* or for further functionality-analyses in 500ul of JNL buffer at pH 7,4 with Theophyllin.

### RNA isolation from purified platelets/cDNA synthesis

TRIzol reagent *(Thermo Fisher Scientific Inc*., *Waltham*, *Massachusetts*, *USA)* was used according to manufacturers protocol to isolate RNA from purified platelets. After isolation RNA quantity was measured using full-spectrum, UV-Vis spectrophotometer (NanoDrop 2000, *Thermo Fisher Scientific Inc*., *Waltham*, *Massachusetts*, *USA*) and diluted to match 1ug/ ul.

Transcription First Strand cDNA Synthesis Kit *(Roche Applied Science*, *82377 Penzberg Bavaria*, *Germany)* was used to generate cDNA from respectively 1ug RNA according to manufacturers guidelines. All primers were designed using Primer-Blast and acquired from Eurofins *(Eurofins Genomics GmbH Ebersberg Germany)*. The sequences of the primers were as follows (forward, reverse): GGGTTGAAGCACTGGACAAT, CAGAGAAGCCTGATTGGAGG for TLR-2; TGAGCAGTCGTGCTGGTATC, CAGGGCTTTTCTGAGTCGTC for TLR-4; ACAACAACATCCACAGCCAA, CTCAGGCCTTGGAAGAAGTG for TLR-9; CTC GCC TTT GCC GAT CCG CC, ACA TGC CGG AGC CGT TGT CG for b-Actin;

### Purity assessment of isolated platelets

For microscopic purity visualization an Axioplan 2 microscope *(Carl Zeiss Microscopy GmbH*, *07743 Jena*, *Germany)* was used. One drop of the purified platelets suspended in 500ul JNL buffer with Theophyllin was spread on a glass plate, covered with a coverslip and allowed to settle for 5 minutes. Analysis was performed using Zeiss Axio Vision Software *(Carl Zeiss Microscopy GmbH*, *07743 Jena*, *Germany)*.

To assess overall cDNA preparation quality exemplary electrophoresis on a 2% Agarose denaturing gel was performed using CD45-staining as a marker for leukocyte contamination and CD-41 referring to platelet glycoprotein IIb/IIIa membrane protein receptor complex as platelet specific marker. Samples were run at 80 Volts for 2 hours and visualized using Molecular Imager® Gel Doc^™^ *(Bio-Rad Laboratories*, *Inc*., *Hercules*, *California*, *USA)* and analysed using Vision Capt Software 14.1 *(Bio-Rad Laboratories*, *Inc*., *Hercules*, *California*, *USA)*. The PCR reaction was run for 30 and 35 cycles respectively with primer specific annealing temperature according to manufacturer guidelines.

Finally exemplary purified platelets samples were double stained with CD-45-PE labelled and CD41-FITC labelled antibodies *(Thermo Fisher Scientific Inc*., *Waltham*, *Massachusetts*, *USA)* and assessed via flow cytometry using a FACS Calibure *(Becton*, *Dickinson and Company*, *Franklin Lakes*, *New Jersey*, *U*.*S*.*)*. A total of 100 000 events were assessed per sample.

### Absolut quantification of copy number

All primers were designed using Primer-Blast and were acquired from Eurofins MWG Operon in 85560 Ebersberg Germany. The sequences of the primers were as follows (forward, reverse): GGGTTGAAGCACTGGACAAT, CAGAGAAGCCTGATTGGAGG for TLR-2; TGAGCAGTCGTGCTGGTATC, CAGGGCTTTTCTGAGTCGTC for TLR-4; ACAACAACATCCACAGCCAA, CTCAGGCCTTGGAAGAAGTG for TLR-9; CTC GCC TTT GCC GAT CCG CC, ACA TGC CGG AGC CGT TGT CG for b-Actin.

All chemicals and fluids used for RNA processing were RNAse and DNAse free. Filtered tips and pre-lubricated tubes were used and all applied materials where treated with RNAse Away (Si*gma-Aldrich Corporation*, *St*. *Louis*, *Missouri*, *United States*) in respect to the high risk of degradation.

We used SYBR® Green *(Thermo Fisher Scientific Inc*., *Waltham*, *Massachusetts*, *USA)* dye–based qPCR to measure DNA amplification. SYBR® Green dye binds any double-stranded DNA generated during PCR and fluoresces only when bound to double-stranded DNA. To measure fluorescence emission during PCR cycles we used a 384 plate-based LightCycler 480 *(F*. *Hoffmann-La Roche AG*, *Basel*, *Schweiz)*. 50ng of generated cDNA were applied in our qPCR protocol. Samples were run according to the following protocol:

The reaction mix for further use was pipetted in 0,2ml tubes (STARLAB International GmbH, Hamburg, Germany) using low binding SafeSeal-Tips (Biozym Scientific GmbH, Hessisch Oldendorf, Germany). It composed of 12,5μl GoTaq® Green Master Mix (Promega Corporation, Wisconsin, United States), equally 10 μM respectively 1μl forward and reverse primers (outlined under cDNA synthesis), 50ng cDNA diluted in 2 μl nuclease free water and finally 8,5μl nuclease free water.

Each sample was assayed in duplicates. The plate was centrifuged (1 min at 2000g at 4°C) and the following thermal protocol used: 10 min at 95°C; 40 cycles of 15 s at 95°C; 60 s at 60°C and hold at 4°C.

To generate a reproducible and reliable standard curve for absolute quantification of mRNA expression using qPCR we used the pGEM®-T Easy Vector System *(Promega Corporation*, *Fitchburg*, *WI*, *USA)* to ligate the corresponding DNA sequence into the provided plasmid with subsequent replication of the plasmid by transformation into JM109 competent bacteria. The afterwards again extracted plasmids were send to Eurofins *(Eurofins Genomics GmbH Ebersberg Germany)* for sequencing to reconfirm correct ligation of the target DNA into the plasmid. The standards were obtained by serial dilution of the dissolved plasmids. Beta-Actin was used as an endogenous control gene to normalise all results.

#### Enzyme-linked immunosorbent assay (ELISA) to detect soluble TLR2 levels in blood-plasma

The human Toll-like receptor 2 (TLR2) ELISA kit from Cusabio (1018 Houston, TX 77036, USA; catalogue number: CSB-E11781h) was used to determine plasma levels of TLR 2 in included patients. Blood-plasma was collected using EDTA as an anticoagulant, centrifuged for 15 minutes at 1000 x g at 4 degrees C° within 30 minutes of collection according to manufacturer’s recommendations guidelines. Levels of TLR2 protein were determined following the manufacturers assay procedure and put into relation using an enclosed standard.

### Statistical analysis

Light Cycler runs were analysed by LightCycler® 480 Software, Version 1.5 *(F*. *Hoffmann-La Roche AG*, *Basel*, *Schweiz)*.

The primary objective of this study was the analysis of TLR 2, 4 and 9 mRNA expression in platelets as well as sTLR2 protein-levels in serum of include patients. On this basis, a total of 54 patients were enrolled in this study for analysis.

Continuous patient data were compared using a T-test or one-way ANOVA if found to follow a Gaussian distribution with Bonferroni's correction for multiple comparisons, otherwise data underwent a Mann–Whitney U-test or Kruskal-Wallis test with Dunn's correction. Categorical differences between patient groups were compared using a Chi-square analysis. A p-value of ≤ 0.05 was considered statistically significant for all analyses. Continuous variables are presented as mean ± standard deviation (SD) if found to follow a Gaussian distribution according to the D'Agosstino-Pearson omnibus normality test, or as median ± lower and upper quartiles if found to follow a non-Gaussian distribution. Categorical patient characteristics are presented as percentages.

All analyses were performed using Graph Pad Prism Version 6.0 (Prism 6 for Mac OS X; GraphPad Software, Inc., La Jolla, CA).

## Results

### Patient characteristics

Baseline Characteristics of the study population are expressed in *Table 1*.

**Table 1:** Patients characteristics. cBaseline Characteristics of the study population.

Characteristics	STEMI	NSTEMI	Non-CAD	p-value
**Gender (Male/Female in %)**	69/31	79/21	64/36	0,7476
**Age (years)**	62±13	67±12	53±11	**0,0153**
**Smokers (%)**	50	42	21	**0,0194**
**Hypertension (%)**	65	93	36	**0,0004**
**Diabetes (%)**	11	42	0	**0,0067**
**BMI**	26±5	30±4	26±4	0,1133
**Thrombocytes (K/μl)**	251±76	242±78	239±47	0,7800
**Leukocytes (K/μl)**	11,5±4,7	9,6±2,85	7,6±1,3	**0,001**
**Haemoglobin (g/dl)**	13,7±1,81	12,8±2,2	14,4±1,5	**0,0106**
**Creatinkinase (U/l)**	1565±1941	317±377	121±70	**<0,0001**
**Troponin T (ng/ml)**	2,547±3,27	0,546±0,82	0,006±0,004	**0,0215**
**CRP (mg/l)**	22,3±41,4	22,3±25,7	5,5±5,6	**0,0052**
**Involved Vessels 0/1/2/3 (%)**	(0/42/38/19)	(0/15/42/43)	(14/0/0/0)	**<0,0001**

Assessed baseline characteristics of enrolled patients included Gender, Age, Smoker or not, Hypertension, Diabetes, BMI, Thrombocytes, Leukocytes, Haemoglobin, Creatininekinase, Troponin T, CRP and number of coronary Vessels involved.

The average age across the entire patient population was 62,63 (SD ± 13,5) years. The age range in the Non-CAD group was 32 to 77 years (53,47 (SD ± 11,55)); in the STEMI group it was 37 to 88 (62,15 (SD ± 13,49)) years and in the NSTEMI group the age range was 45 to 80 (67,21 (SD ± 11,62)) years. The STEMI population was composed of 69% men, in comparison to 79% men in NSTEMIs and 64% men in the CAD excluded group.

While there were, in line with the basic characteristic of ACS-patients, statistically significant differences in age, cardiovascular risk factors (Diabetes Mellitus, history of or current use of cigarettes, hypertension), cardiac cell death markers (Troponin t, Creatininekinase), haemoglobin and infection-markers (CRP, Leukocytes), there were no differences in gender, body weight and thrombocytes.

### TLR2-mRNA expression in isolated platelets

Analysis of the RT-qPCR data revealed an absolute copy number of TLR2 mRNA of 4,818e^-005^ (SD ± 2,179e^-005^) copies/μl in our Non-CAD-group.

When compared to the STEMI and NSTEMI patients there was a statistically significant difference between groups as determined by Kruskal-Wallis test *(p = 0*,*01)*. The Dunn´s multiple comparison test showed a statistically significant difference between Non-CAD and NSTEMI/STEMI patients (p = 0,02/p = 0,04).

Platelets isolated from patients suffering from NSTEMI presented with a TLR2 mRNA expression of 13,34e^-005^ (SD ± 2,564e^-005^) copies/μl while patients with STEMI presented with 7,950e^-005^ (IQR 4,983e^-005^–11,23 e^-005^) copies/μl.

**Fig 1:**
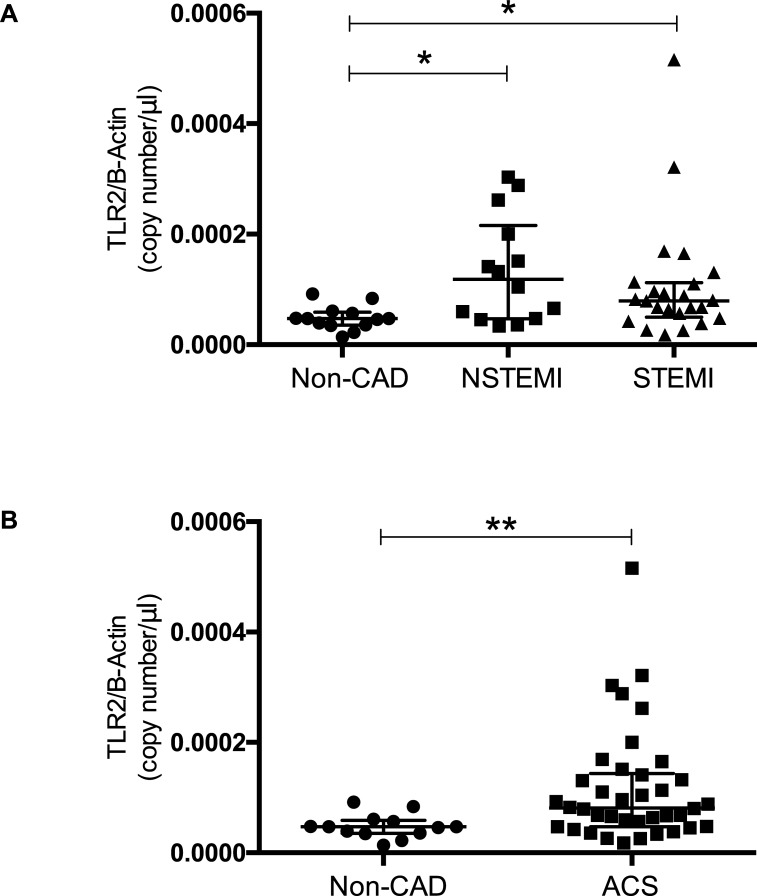
TLR2 mRNA expression in isolated platelets. TLR2 mRNA expression levels in platelets isolated from patients with ACS and in patients with via percutaneous interventional coronarangiography excluded coronary artery disease in total **(B)** and in a subgroup analysis differentiating in-between STEMI and NSTEMI accordingly **(A)**. Data are presented as scatter plots with median and interquartile range *p<0,05, **p<0,001.

There was no statistical relevant difference in platelet TLR2 mRNA-expression of NSTEMI and STEMI-patients. (Fig 1A)

Patients suffering from AMI presented with an overall elevated expression of TLR2 when compared to patients included in our non-CAD-group (8,105 e^-005^ (IQR 4,745 e^-005^–14,35e^-005^) vs. 4,818 e^-005^ (SD ± 0,6045e^-005^)) copies/μl; p = 0,0033). (Fig 1B)

### Soluble TLR2 protein plasma levels

**Fig 2:**
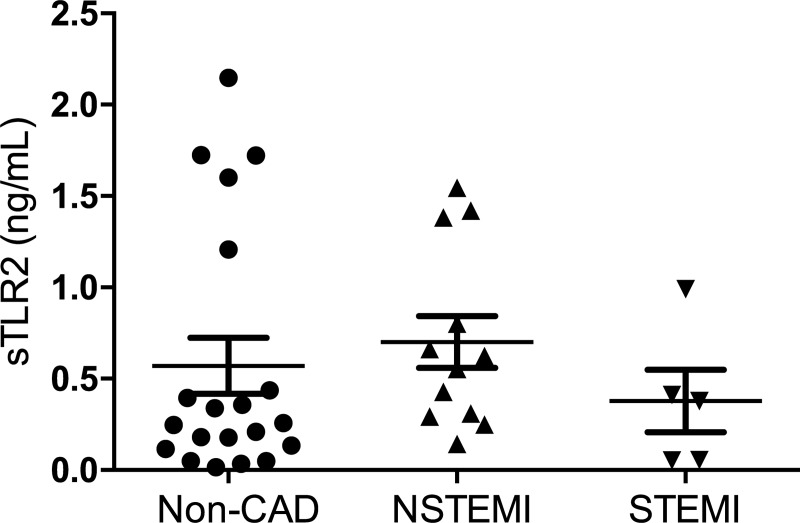
Plasma expression levels of soluble TLR 2. Plasma expression levels of soluble TLR 2 as measured in ELISA in patients with ACS (NSTEMI, STEMI) and in patients with via percutaneous interventional coronarangiography excluded coronary artery disease. Data are presented as box plots with median and SEM.

There was no evident statistically significant alteration in-between plasma levels of sTLR in patients with NSTEMI, STEMI and non-CAD as is shown in Fig 2 Non-CAD patients presented with a TLR2-plasma-level of 0,252 (IQR 0,12–1,015) ng/ml). NSTEMI 0,7005 ng/ml (SD ± 0,49) ng/ml and STEMI patients 0,378 ng/ml (IQR 0,055–0,0701) had fairly similar TLR2-plasma levels, all in all not statistically significant amongst themselves and when compared to non-CAD levels of TLR2 plasma levels (p = 0,1946; p = >0,9999; p = 0,1230).

### TLR4-mRNA expression in isolated platelets

**Fig 3:**
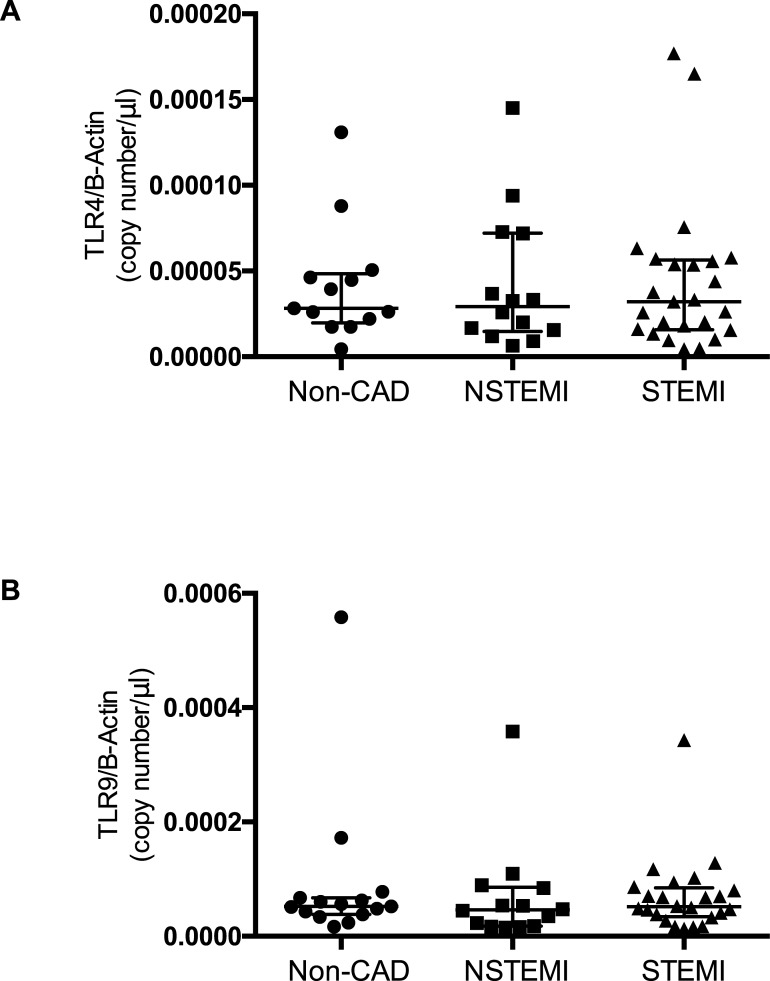
TLR4 and -9 mRNA expression in plates. TLR4 **(A)** and TLR9 **(B)** mRNA expression levels in platelets isolated from patients with ACS and in patients with via percutaneous interventional coronarangiography excluded coronary artery disease Data are presented as scatter plots with median and interquartile range.

There was no statistical significant difference in TLR4 mRNA expression in included patients (Fig 3). Kruskal-Wallis test and Dunn's multiple comparisons test showed no difference between the individual groups *(p = 0*,*6822)*. Subjects included in the Non-CAD-group had 2,830e^-005^ (IQR 1,970e^-005^–4,835e^-005^) copies/μl after normalisation with beta-Actin while patients with NSTEMI and STEMI presented with comparable amounts of mRNA (2,925e^-005^ (IQR1,468e^-005^–7,213e^-005^); 3,210e^-005^ (IQR 1,580e^-005^–5,635e^-005^)).

### TLR9-mRNA expression in isolated platelets

There was no statistical significant difference in TLR9 mRNA expression in included patients. Kruskal-Wallis and Dunn's multiple comparisons test showed no difference between the individual groups *(p = 0*,*3811)*. Subjects included in the Non-CAD-group presented with an absolute copy number from TLR9 mRNA of 5,230e^-005^ (IQR 3,830e^-005^–6,700e^-005^) copies/μl after normalisation with beta-Actin, while patients with NSTEMI and STEMI presented with comparable amounts of mRNA (4,610e^-005^ (IQR 1,755e^-005^–8,560e^-005^); 5,150e^-005^ (IQR 3,400e^-005^–8,458e^-005^)).

## Discussion

We aimed to determine whether a differential expression of mRNA coding for receptors of the innate immune system, that recently have been described in platelets, does occur in patients with acute myocardial infarction (AMI). For this purpose, the mRNA coding TLR-2, TLR-4 and TLR-9 were quantified in highly purified platelets from patients with AMI.

We hypothesized that there is a distinct alteration in platelet immunoreceptor mRNA levels possibly consistent with a systemic response or trigger of plaque destabilisation in AMI. [60]

Using absolute plasmid based quantitative Real Time PCR we investigated TLR-mRNA expression of platelets, purified from peripheral blood, in patients with STEMI, NSTEMI and patients with rule out of coronary artery disease by coronary angiography (non-CAD).

Since purity and integrity of isolated platelets is crucial to this study we paid special attention to it by including purity assessment of isolated platelets via PCR-analysis, microscopy and flow cytometry.

Toll like receptors are part of the superfamily of pattern recognition receptors eliciting the innate immune response in inflammation. [61] Several studies show that PAMPs or DAMPs mediated TLR2-, TLR4- and TLR9- activation impels prothrombotic platelet characteristics. [22, 53] Also local inflammation, fuelled by the partly TLR-mediated immune response at atherosclerotic lesions sites, promotes fibrous cap rupture and erosion in plaque formations, ultimately leading to AMI. [24]

When activated, platelets significantly increase the splicing and translation to protein of cytosolic megakaryocyte derived mRNA.[15] Some of this platelet mRNA encodes for the TLR receptors themselves. [62]

Many studies illustrate the importance of different TLR signalling-pathways in ACS and platelet activation. [2, 8] There is evidence of on-site TLR over-expression for example in monocytes within a coronary thrombus and in atherosclerotic plaque formations. [36, 63] Recent studies also describe alteration in systemic platelet TLR expression in ACS and stable angina pectoris patients rendering platelet TLR-expression a possible marker or target in ACS diagnostics. [64]

Whether alteration in TLR receptor expression is a mainly systemic response or a local phenomenon is yet to be established.

Results of our study show up-regulation of TLR 2 mRNA expression in platelets isolated from patients suffering from STEMI and NSTEMI when compared to non-CAD group. This correlates with recent findings of enhanced platelet TLR expression detected by flow cytometry in patients with acute coronary syndrome. [64]

As was proven many times beforehand, when studies e.g. linked systemic infection to higher rates of ACS, systemic alteration can trigger an exaggerated local response and hence result in progression of atherosclerotic lesions and plaque instability and ultimately in ACS. [65, 66]

Our results suggest an involvement of systemic platelet TLR-2 mRNA expression in ACS. This, to the best of our knowledge, is the first time that up-regulated platelet specific TLR-2 mRNA expression is described in patients with ACS.

This allows two different approaches, drawing a fundamentally variable picture of ACS-pathogenesis.

First of all, elevated TLR-2 mRNA expression could be a result of altered megakaryocytic phenotypes, more precisely, increased TLR-2 mRNA expression in bone marrow- or peripheral-blood-megakaryocytes and consequently increased transmission to produced platelets.

While given that TLR2 promotes immune-mediated plaque instability, atherosclerosis and exerts pro-thrombotic abilities, this would suggest a regimented chain of events leading to ACS rather than an acute event. [22, 53] This is supported in part by our experimental design in which sample collection took place in a 24 hours time frame following ACS. Considering platelet life-time-span, 8–9 days would allow for a complete platelet turnover. [67] Consequently 24 hours would not be enough to significantly change platelet features.

On the other hand, many studies implicate induced systemic inflammatory markers in the pathogenesis of ACS. [68] Platelets, as part of the innate immune response, are interwoven with the anti-inflammatory response not at last via activation through TLRs as stated before. [22, 31, 41, 42] Hence, induced platelet mRNA expression of TLR 2 could also be a result of induced splicing of megakaryocytic pre-mRNA maybe even fuelled, in the sense of a positive feedback-loop, by TLR-receptor activation. [15, 16, 18] Basic characteristics of our included patients support this thesis since included patients with ACS presented with higher levels of markers for inflammation.

Both possibilities would render platelet TLR2 mRNA expression a promising marker in ACS diagnosis and therapy. The link between both theories is supported by recent findings in mice-models, showing that inflammation, through TLR2, can increase maturation and modulate the phenotype of megakaryocytes. [69]

Recently, studies emphasise the influence of plasma decoy receptors in TLR receptor regulation. [57] Monocytes, reportedly, upon stimulation release a modified TLR2-receptor -sTLR2- which is believed to suppress TLR-activation. However, the exact mechanisms of sTLR function are yet to be discovered. Using ELISA we quantified plasma levels of sTLR2 in NSTEMI, STEMI and non-CAD patients. There was no significant difference in plasma levels of sTLR2, which correlates with recent findings reporting no significant change of TLR2 expression in monocytes in a similar setting. [50, 63] Whether patients with ACS would profit from a mechanism to prevent an exaggerated inflammatory response via sTLR2 activation is yet to be established.

Absolute copy numbers of TLR2, TLR4 and TLR9 mRNA in platelets were low. This was expected since recent studies entitle platelets to contain not more than a total of 2x10^-15^ g mRNA per platelet. [11]

Analysis of our qPCR data showed no statistical difference in platelet TLR4 and TLR 9 mRNA expression in patients with ACS when compared patients with coronary artery disease excluded by angiography. Opposed regulation of TLR2 compared to TLR9 and TLR4 mRNA expression patterns has been described before. [70, 71] Apart from the high sequence homology between TLR2 and TLR4 they seem to react to different stimuli and elicit individual stimuli. Our data confirms this hypothesis. In contrast to recent publications, which describe elevated baseline expression of TLR9 protein levels in ACS platelets, our results show no alteration in platelet TLR9 mRNA. [72] This is overall consistent with the fact that platelets, being a-nucleate cells, mainly adjust their protein expression via posttranslational regulation and suggest such modulation.

Apart from several differences in patient characteristics between the 3 included groups (outlined in Table 1) there were no significant differences in TLR4, TLR2 and TLR 9 mRNA expression in patients with coronary artery disease excluded by angiography. This is on the one hand consistent with the theory of a fixed platelet transcriptom and all the more qualifies altered mRNA expression as a reliable marker of disease.

## Limitations

The results of this study support our hypothesis. However, our study has some limitations. The differences in patients characteristics outlined in Table 1 are partly an image of the underlying disease and its risk factors. Therefore included patients suffering from ACS presented with higher levels of troponin-t and creatininekinase. Also risk factors such as diabetes, adipositas, history of or current smoking are higher in patients suffering from ACS. What could interfere with our results is a significant difference in leukocyte and CRP expression in-between the 3 groups since the TLR-family is involved in orchestrating the immune response on pathogens. However, patients presenting with clinical sings of infection and/or being under current antibiotic therapy were excluded from this study.

Further limitation include that we did not investigate TLR protein in platelets or took into account posttranslational modifications. Also follow-up data on influence of platelet TLR mRNA-expression on patient outcome would be interesting as well as over a definite time-course. In addition to that, there is our relatively small sample size, which limits liability of our results. However, a strength of our study is the special attention that we payed to the purity of our isolated platelets, thereby excluding contamination by leukocyte mRNA. In this context: We did not evaluate general platelet activation in included patients e.g. by analysing platelet aggregation or Pselectin.

Also TLR expression in platelets could be subject to rapid fluctuation and therefore the different time points of sample collection, although being within 24hours, could affect our results. Finally, the validity of analysis of plasma levels of sTLR2 is limited through a variance in sample number due to technical storage related failure.

## Conclusion

To the best of our knowledge, we describe for the first time elevated TLR2 mRNA expression in platelets of patients with ACS compared to a non-CAD group. This suggests platelet TLR2 mRNA expression as an alternative platelet activation pathway in ACS and singles out platelet TLR2 mRNA as a potential marker and potential target for in therapy and diagnosis. Our data warrants further validation in larger clinical trials.

## Supporting information

S1 Table Collected set of laboratory data of included patients. (XLSX)

S2 Table Purity assessment of samples using NanoDrop^™^. (XLSX)

S3 Table Absolute copy numbers after quantification using real-time PCR. (XLSX)

S4 Table Results of sTLR 2 Enzyme-linked immunosorbent assay. (XLSX)

## Abbreviations

ACS: Acute Coronary Syndrome
BM: Bone Marrow
RNA: Ribonucleic Acid
PAMPS: Pathogen-associated molecular patterns
DAMPS: Damage-associated molecular patterns
TLR: Toll-like-receptors
I/R injury: Ischemia/reperfusion injury
sTLR: Soluble Toll like receptor
ELISA: Enzyme-linked immunosorbent assay
PRP: Platelet rich plasma
qPCR: Real-time quantitative PCR
